# Mental fatigue is linked with attentional bias for sad stimuli

**DOI:** 10.1038/s41598-019-45428-0

**Published:** 2019-06-19

**Authors:** Kyosuke Watanabe, Akihiro T. Sasaki, Kanako Tajima, Kenji Mizuseki, Kei Mizuno, Yasuyoshi Watanabe

**Affiliations:** 1 Department of Physiology, Osaka City University Graduate School of Medicine, Osaka, 545-8585 Japan; 2 Pathophysiological and Health Science Team, Division of Bio-Function Dynamics Imaging, RIKEN Center for Life Science Technologies, Hyogo, 650-0047 Japan; 3 Laboratory for Pathophysiological and Health Science, RIKEN Center for Biosystems Dynamics Research, Hyogo, 650-0047 Japan; 4 The ‘Compass to Healthy Life’ Research Complex Program, RIKEN, Hyogo, 650-0047 Japan

**Keywords:** Human behaviour, Preclinical research

## Abstract

Previous studies have revealed that patients with chronic fatigue syndrome and affective disorders (such as depression and anxiety disorders) exhibit a vigilant attentional bias toward negative emotional stimuli. However, it remains unclear whether the change in an attentional bias for negative emotional stimuli can be induced by mental fatigue in healthy individuals. To address this question, we examined healthy participants’ (n = 27) performance in a visual probe task and emotional Stroop task before and after the mental-fatigue-inducing task. We demonstrated that acute mental fatigue induced by the long-lasting working memory task led to the alteration of cognitive processing of negative emotional information in the healthy volunteers.

## Introduction

Fatigue is characterized by inefficiency in mental or physical activities and is commonly experienced in everyday modern life; large community surveys have reported that up to half of the general adult population complains of fatigue^1,2^. In Japan, it is even more common, with more than half of the population reporting fatigue. Of these, more than one-third experienced chronic fatigue lasting for over 6 months^3^. Acute fatigue is a physiological phenomenon that attenuates after a period of rest; chronic fatigue, on the other hand, is caused by the accumulation of acute fatigue^4^. Chronic fatigue could be considered as an intermediate state between healthy and clinical states. Chronic fatigue syndrome (CFS), which is now also known as Myalgic Encephalomyelitis/Chronic Fatigue Syndrome, refers to a clinical state distinguished by severe disabling fatigue and various symptoms such as impairments in concentration, memory, or sleep^5^. Though the figures vary among different countries, the average worldwide prevalence of chronic fatigue is 10%, and that of CFS is 1%^6^. Fatigue has considerable costs for both individuals and society; therefore, there is a need to establish methods for coping with this widespread problem.

Patients with CFS are known to have a higher prevalence of comorbid psychiatric disorders such as depression or anxiety disorder. Afari and Buchwald noted in their review article^7^ that 50–75% of patients with CFS had a lifetime history of major depressive disorder (MDD), while 17–25% and 2–30% had lifetime histories of panic disorder and generalized anxiety disorder, respectively. In Japan, Matsuda *et al*.^8^ reported that 26% and 7% of patients with CFS had lifetime comorbid MDD and panic disorder, respectively. These are higher than the estimates of the lifetime prevalence of the general adult population (MDD: 16.6%, panic disorder: 4.7%, generalized anxiety disorder: 5.7%)^9^. Fatigue is a common symptom of depression and certain anxiety disorders, such as generalized anxiety disorder, and is in fact included in the diagnostic criteria for these disorders in the *Diagnostic and statistical manual of mental disorders, fifth edition*^10^. Accordingly, fatigue and affective disorders such as depression or anxiety may be intrinsically linked.

Beck and his colleagues proposed a cognitive model of depression and anxiety^11,12^, wherein they advocated that ‘negative schema activation’ plays a key role in the development and maintenance of depressive and anxiety symptoms. Schemas are defined as cognitive frameworks enabling us to process stimuli, assign them meaning, and determine how to interpret our experiences. Activation of negative schema by genetic and personality vulnerability and environmental triggers leads biased attention, memory and processing^12^. Healthy individuals can generally cope with negative emotional stimuli by balancing top-down cognitive control with bottom-up cognitive processing. Top-down cognitive control is an explicit and strategic form of regulatory processing; bottom-up cognitive processing is an implicit and automatic processing^11,13^. In contrast to the case of healthy individuals, sustained negative schema activation in symptomatic individuals induces a loss of the balance and can lead to ineffective coping, avoidance, and finally the occurrence of depressive and anxiety symptoms.

To determine which the information entering the sensory system is processed, attentional bias plays an important role. Attention can be influenced by a variety of processes, such as exogenous (bottom-up) attention, endogenous (top-down) attention, or emotional attention^14–16^ (for review: Pool *et al*.^17^). Exogenous attention is stimulus-driven, rapid, and involuntary type of attention, whereas endogenous attention is goal-directed, less rapid than exogenous attention, and voluntary type of attention. Emotional attention is affective-driven, rapid, involuntary (like exogenous attention), and is influenced by individual’s affective state^18^. These three systems can operate independently and simultaneously, and interact with one another^16^. Numerous studies have revealed that patients with depression (e.g., Gotlib *et al*.^19^; Mitterschiffthaler *et al*.^20^) or anxiety (e.g., Becker *et al*.^21^; Mogg *et al*.^22^) show a vigilant attentional bias toward negative emotional stimuli, which, according to Beck’s cognitive model, is induced by negative schema activation. Similarly, patients with CFS exhibit a vigilant attentional bias toward health-threatening information^23^. Winer and Salem noted in their review article^24^ that patients with depression or anxiety showed not only a vigilant attentional bias toward negative emotional stimuli, but also an avoidant attentional bias for positive emotional stimuli. To measure attentional bias, a lot of studies employed a visual probe task or emotional Stroop task. The visual probe task is designed to measure attentional distribution by presenting emotional stimuli to alternative locations on a computer screen. Subsequently, researchers determine how rapidly participants can respond to the probes presented in these locations. Response latencies tend to be shorter when the probe is displayed to attended areas, thereby allowing researchers to determine the degree of attentional focus on emotional stimuli by comparing the latencies across different probe locations. In contrast, during the emotional Stroop task, participants must ignore the content of emotional words while naming the text colour of the word. Since attention to emotional distracters would interfere with participants’ concentration on this task, attentional bias can be gauged by examining the difference in response latencies for emotional and non-emotional words. We used both these tasks in the present study to investigate whether the effect of fatigue on attentional bias varied depending on the task type.

While attentional bias toward negative emotional stimuli is common among patients with affective disorders and CFS, it is still unclear if the change in attentional bias is observed because of mental fatigue among healthy individuals. Therefore, we hypothesised that recoverable (not pathological) fatigue was linked with attentional bias for negative emotional stimuli, and a change in the attentional bias for negative emotional stimuli was observed at the acute mental fatigue state. In the present study, we investigated this hypothesis using the face dot-probe (FDP) task, which is a type of visual probe task, and the emotional Stroop task before and after two-back task, which can induce mental fatigue^25^.

## Results

### Two-back task

Task performance and the results of paired samples *t-*test comparing performance between the first and final blocks are summarized in Table 1. While the mean reaction time of the last block was faster than that of the first block {the first block, 551.1 ± 181.3 (mean ± SD); the last block, 495.1 ± 129.4; *P* = 0.014; *g* (Hedge’s *g*) = 0.306}, the two blocks did not differ in terms of accuracy (*g* = −0.179). These results are consistent with our previous study exploring the use of a 30-min load of the two-back task to induce mental fatigue^25^. While performance on the mental-fatigue-inducing task was not significantly reduced (Table 1), it was previously shown that performance on another fatigue-evaluation task (advanced trail making test) was significantly lower after the mental-fatigue-inducing task than before it^26^, suggesting that acute mental fatigue is caused by attempting to maintain good working memory performance for a prolonged period^25^. Furthermore, the mean visual analogue scale (VAS) scores for fatigue increased as the task progressed, and a paired samples *t-*test revealed that VAS scores in the last block were significantly higher than were those in the first block (the first block, 44.3 ± 19.4; the last block, 71.5 ± 23.9; *P* < 0.001; *g* = −1.188).

**Table 1:** Performance on the two-back task.

	All	First block	Last block	*t*	*df*	*P*
Mean reaction time (ms)	518.0 ± 128.2	551.1 ± 181.3	495.1 ± 129.4	2.653	23	0.014
Mean correct answer rate (%)	77.9 ± 9.9	72.0 ± 17.1	75.0 ± 15.2	−0.818	23	0.422
Mean incorrect answer rate (%)	19.7 ± 9.4	25.2 ± 15.3	21.5 ± 15.0	1.144	23	0.264
Mean unanswered rate (%)	2.5 ± 2.7	2.8 ± 6.1	3.5 ± 7.3	−0.355	23	0.726
Mean VAS score		44.3 ± 19.4	71.5 ± 23.9	−6.535	23	0.000

Values are expressed as mean ± SD. The data from the paired samples *t*-test for each measure are also shown. *df*, degrees of freedom.

### Face dot-probe task

**Figure 1:**
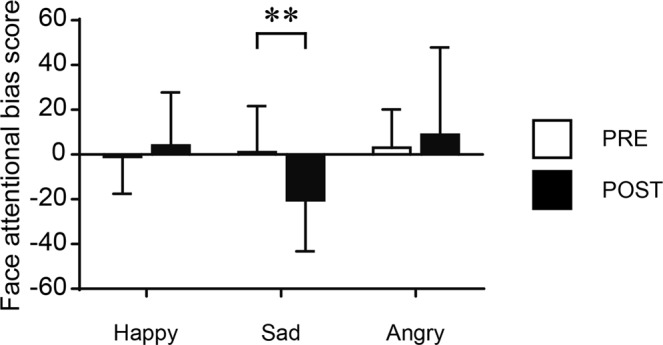
Changes in face attentional bias scores before (PRE) and after (POST) the mental-fatigue-inducing task (n = 23). Values are expressed as mean ± SD. **P < 0.01

Correct answer rate (Pre-fatigue, 99.6 ± 0.85; Post-fatigue, 99.1 ± 1.11) and reaction time (Pre-fatigue, 356.3 ± 48.3; Post-fatigue, 362.5 ± 49.9) were not significantly different between before and after the mental-fatigue-inducing task. Face attentional bias scores of each emotional condition are also shown in Table 2. A two-way (timing [pre-/post-fatigue] × face emotion) repeated-measures analysis of variance (ANOVA) on face attentional bias scores revealed a statistically significant main effect of face emotion {*F*(2, 46) = 4.373, *P* = 0.018, η_p_^2^ (partial eta-squared) = 0.160} and an interaction {*F*(2, 46) = 5.686, *P* = 0.006, η_p_^2^ = 0.198}; the main effect of timing was not significant {*F*(1, 23) = 1.388, *P* = 0.251, η_p_^2^ = 0.057}. Post-hoc multiple comparisons (Bonferroni-corrected) revealed that the bias score for sad faces decreased (Pre-fatigue, 1.25 ± 20.5; Post-fatigue, −20.31 ± 21.6; *P* = 0.001; *g* = 0.969) following the fatigue-inducing session; whereas, the scores for happy (*P* = 0.587, *g* = −0.118) and angry (*P* = 0.499, *g* = 0.185) did not (Fig. 1).

**Table 2:** Performance on the face dot-probe task.

	Pre-fatigue	Post-fatigue
Correct answer rate (%)	99.6 ± 0.85	99.1 ± 1.11
Reaction time (ms)	356.3 ± 48.3	362.5 ± 49.9
Face attentional bias score: Happy	−0.94 ± 16.3	1.64 ± 23.7
Face attentional bias score: Sad	1.25 ± 20.5	−20.31 ± 21.6
Face attentional bias score: Angry	3.60 ± 16.5	9.29 ± 37.3

Values are expressed as mean ± SD.

### Emotional Stroop task

**Figure 2:**
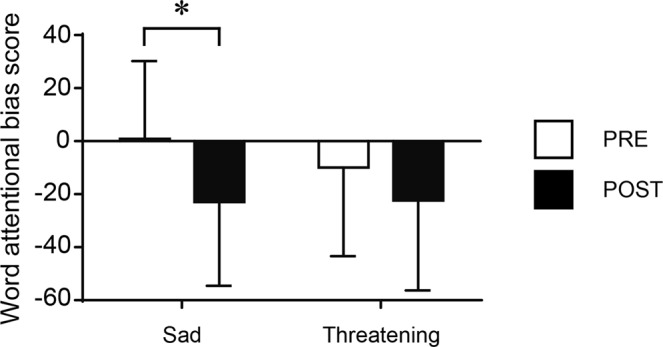
Changes in word attentional bias scores before (PRE) and after (POST) the mental-fatigue-inducing task (n = 23). Values are expressed as mean ± SD. *P < 0.05

Correct answer rate (Pre-fatigue, 97.0 ± 2.11; Post-fatigue, 96.5 ± 3.12) and reaction time (Pre-fatigue, 630.1 ± 96.0; Post-fatigue, 633.9 ± 88.2) were not significantly different between before and after the mental-fatigue-inducing task. Word attentional bias scores of each emotional condition are also shown in Table 3. A two-way (timing × word emotion) repeated measures ANOVA on word attentional bias scores revealed that the main effect of timing showed a trend level of significance {*F*(1, 23) = 3.756, *P = *0.065, η_p_^2^ = 0.140}, whereas neither the main effect of word emotion {*F*(1, 23) = 1.084, *P* = 0.309, η_p_^2^ = 0.045} nor the interaction {*F*(1, 23) = 1.958, *P* = 0.175, η_p_^2^ = 0.078} was significant. Post-hoc multiple comparisons (Bonferroni-corrected) examining the effect of acute mental fatigue on attentional bias revealed that the bias score for sad words decreased (Pre-fatigue, 2.10 ± 29.4; Post-fatigue, −20.73 ± 32.7; *P* = 0.014; *g* = 0.710) after the mental-fatigue-inducing session; however, the bias score for threat words (*P* = 0.269, *g* = 0.357) did not (Fig. 2).

**Table 3:** Performance on the emotional Stroop task.

	Pre-fatigue	Post-fatigue
Correct answer rate (%)	97.0 ± 2.11	96.5 ± 3.12
Reaction time (ms)	630.1 ± 96.0	633.9 ± 88.2
Word attentional bias score: Sad	2.10 ± 29.4	−20.73 ± 32.7
Word attentional bias score: Threat	−8.87 ± 33.1	−21.21 ± 33.7

Values are expressed as mean ± SD.

### Change in the participants’ mood

After the mental-fatigue-inducing task session, VAS scores of fatigue (Pre-fatigue, 34.8 ± 22.2; Post-fatigue, 64.9 ± 24.7; *P* = 0.000; *g* = −1.229) and depression (Pre-fatigue, 14.7 ± 16.1; Post-fatigue, 24.7 ± 25.3; *P* = 0.030; *g* = −0.433) significantly increased, and that of motivation (Pre-fatigue, 54.7 ± 21.0; Post-fatigue, 43.0 ± 23.8; *P* = 0.014; *g* = 0.501) significantly decreased. Meanwhile, the changes in VAS score of anxiety (Pre-fatigue, 18.8 ± 15.5; Post-fatigue, 24.0 ± 23.3; *P* = 0.218; *g* = −0.245) was not statistically significant, and that of sleepiness (Pre-fatigue, 43.3 ± 22.3; Post-fatigue, 52.2 ± 29.9; *P* = 0.087; *g* = −0.318) showed a trend level of significance (Table 4).

**Table 4:** Changes in the participants’ mood.

	Pre-fatigue	Post-fatigue	*t*	*df*	*P*
Fatigue	34.8 ± 22.2	64.9 ± 24.7	−7.802	23	0.000
Depression	14.7 ± 16.1	24.7 ± 25.3	−2.319	23	0.030
Anxiety	18.8 ± 15.5	24.0 ± 23.3	−1.267	23	0.218
Motivation	54.7 ± 21.0	43.0 ± 23.8	2.666	23	0.014
Sleepiness	43.3 ± 22.3	52.2 ± 29.9	−1.787	23	0.087

Values are expressed as mean ± SD. The data from the paired samples *t*-test for each measure are also shown. *df*, degrees of freedom.

## Discussion

The principal finding of this study is that healthy individuals developed an avoidant attentional bias for sad stimuli after they were made to experience mental fatigue. At first glance, our results are somewhat at odds with previous findings reporting that patients with clinical depression showed a vigilant attentional bias toward sad stimuli and an avoidant attentional bias for positive stimuli^24^. We can interpret this finding as follows: healthy individuals can successfully engage in top-down regulation to concentrate on the main task (effective coping) when not fatigued. On the other hand, when experiencing acute mental fatigue, it becomes difficult to sustain this top-down regulation in the same way, which forces them to change their behavioural strategy to avoid sad stimuli (ineffective coping). In other words, cognitive resources for top-down control become relatively insufficient due to acute mental fatigue, so that participants need to change their behaviour (avoiding sad stimuli) in order to adapt the situation and manage to finish the task. After the mental-fatigue-inducing task, the subjective level of depression and fatigue increased and that of motivation decreased, whereas that of anxiety was not changed (Table 4). As the result of the mood deterioration, more amounts of cognitive resources should be required to process sad emotional information, so that they become unable to efficiently cope with sad (but not threatening) emotional information. Both the shortage of cognitive resources of top-down regulation and increased demand of resources to process sad emotional information might cause the behavioural change. In the case of the face dot-probe task, this change is observed as the slower reaction time under the congruent condition than incongruent condition, meanwhile, in the case of the emotional Stroop task, the behavioural change is found as faster reaction to the colour of the sad word than neutral word.

Frontoparietal networks (e.g., a network between frontal eye field and intraparietal sulcus/superior parietal lobule) are involved in the top-down control of visuospatial attention^14^. Tanaka *et al*.^27^ found that prolonged mental fatigue induced a change in the activation of the prefrontal cortex. Mizuno *et al*.^28^ revealed that patients with childhood chronic fatigue syndrome showed an overactivity of prefrontal regions during attention control, and healthy children and adolescents also exhibited an overactivity of inferior frontal gyrus during a state of mental fatigue. These findings suggest that mental fatigue increases the consumption of cognitive resources for the main task, which would induce an altered pattern of top-down control (endogenous attention). To verify this hypothesis, we intend to conduct a functional magnetic resonance imaging study in the near future.

We used both a face dot-probe task and an emotional Stroop task because we intended to examine how the difference in task type influences the effect of fatigue on attentional bias. The results showed that individuals experiencing acute mental fatigue exhibited a change in attentional bias only for sad stimuli but not for threatening stimuli, regardless of the task type. The medium-to-large effect sizes of these changes in attentional bias were observed (see Results). The change in the subjective level of depression (but no change in anxiety) is thought to be the reason why the change in the attentional bias occurs only for sad stimuli but not for threatening stimuli. In the previous studies, patients with depression showed an attentional bias toward sad stimuli, whereas patients with anxiety exhibited a bias toward threatening stimuli. CFS is more frequently accompanied by depression than by anxiety disorders^7,8^. Considering these facts together, fatigue might have a greater impact on attentional bias for sad stimuli than for threatening stimuli because of its stronger link with depression than anxiety disorders.

The finding that acute mental fatigue induces an avoidant attentional bias for sad stimuli even for healthy individuals implies that mental fatigue has the potential to induce an alteration of cognitive processing of negative emotional information. According to the Beck’s cognitive model, negative schema activation, which leads an attentional bias for emotional stimuli, induces avoidance from negative emotional information and also ineffective coping, resulting in the symptoms of depression^11^; that is, the avoidance from negative emotional information at the fatigued state might be a prelude to the clinical stage. Although there are numerous treatments for depression, such as pharmacotherapy or cognitive therapy^29–31^, there are relatively few preventive methods. A particularly relevant area for our findings is attentional bias modification training, which has proven useful for reducing the symptoms of depression^32–34^ and anxiety^35–37^. However, it remains unclear whether attentional bias modification training is applicable to prevention. Further research is therefore necessary to determine whether prevention of fatigue is effective for preventing the development of psychopathology.

In conclusion, an avoidant attentional bias for sad stimuli was observed among individuals experiencing acute mental fatigue. We believe that this finding will add a new perspective to our understanding of the relationship between an attentional bias for negative emotional stimuli and fatigue, and could give us the insight into the psychopathology of fatigue-related and affective disorders. However, there is still a gap between our results obtained from healthy individuals with acute mental fatigue and previous results obtained from patients with pathological fatigue^23^. We examined the former in this study and found an avoidant attentional bias. In contrast, Hou *et al*.^23^ examined the latter, and observed a vigilant attentional bias. To bridge the gap and clarify the psychopathology of the abovementioned disorders, we intend to conduct further research on individuals with chronic fatigue in the near future.

## Methods

### Participants

Twenty-seven healthy individuals (13 males and 14 females, mean ± SD age = 41.4 ± 8.3 years) participated in this study. All participants had normal or correct-to-normal visual acuity and met the following inclusion criteria: (1) 20–60 years old, (2) no history of past or present psychiatric disorders, and (3) no work-related difficulties because of fatigue. Participants’ fatigue level was assessed using Chalder’s fatigue scale^38,39^, and their average score was 1.9 ± 2.5 (11-item, bimodal scoring). The study protocol was approved by the Ethics Committee of RIKEN Center for Life Science Technologies (RIKEN-Kobe2-IRB-17-64) and the Osaka City University Center for Health Science Innovation (OCU-CHSI-IRB No. 15). This experiment was conducted in accordance with the Declaration of Helsinki, and all participants gave their written informed consent before participation.

### Experimental setup

A laptop computer (Lenovo B50; Lenovo Japan Co., Tokyo, Japan; display resolution: 1024 × 768 pixels) was used to run all the experimental tasks. The tasks were controlled via the Presentation® software (Version 18.1; Neurobehavioral Systems Inc., Berkeley, CA, USA).

### Procedure

About one week before the experiment was conducted, all the tasks were explained and participants practiced these tasks. Participants performed an emotional task session before and after a mental-fatigue-inducing task session. The emotional task session included the FDP and emotional Stroop tasks, whereas the mental-fatigue-inducing task session included the two-back task. The two-back task requires the use of working memory^40^, and has been previously used to induce acute mental fatigue^41–44^. The subjective levels of fatigue, depression, anxiety, motivation, and sleepiness were assessed using VAS before the pre- and post-emotional task session. VAS is a psychometric response scale used to measure subjective feelings, and the position of the participants’ response is assigned a score from 0 to 100.

### Experimental tasks and stimuli

#### Two-back task

In the mental-fatigue-inducing task session, participants performed a two-back task, which required them to view consecutively presented numerals randomly selected from among the numerals 1, 2, 3, or 4. They were asked to determine whether each newly presented character was identical to that presented two trials before by pressing the corresponding button with one of their index fingers. The stimulus presentation was as follows: after a fixation cross was displayed for 1000 ms, numbers were displayed for 500 ms. One block comprised 20 trials (30 seconds), and 56 blocks were performed. Participants were given 18-s intervals between blocks, during which they specified their subjective level of fatigue via VAS. The entire duration of the two-back task was approximately 45 minutes.

#### Face dot-probe task

The facial stimuli comprised pictures of human faces taken from the Advanced Telecommunications Research (ATR) facial expression image database (DB99, ATR-promotions, 2006). We selected 10 each of happy, sad, and angry faces, and then paired them with neutral faces of the same actor.

**Figure 3:**
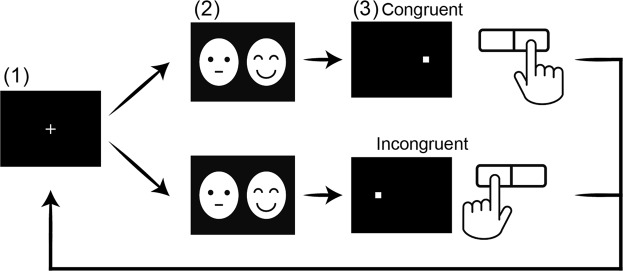
Face dot-probe task procedure. (1) A white fixation cross was presented at the centre of the screen for 500 ms. (2) A neutral face picture paired with an emotional face picture was presented for 500 ms. (3) A white dot was displayed in the same location as one of the faces. Participants pressed a button corresponding to the side where the dot was displayed using their index finger as quickly as possible.

Each trial began with a white fixation cross presented at the centre of the screen for 500 ms; this was followed by a pair of face pictures (434 × 329 pixels; x = ±300 pixels from the centre, y = 0 pixels from the centre) displayed bilaterally for 500 ms. After the face pair disappeared, a white dot appeared in the location of one of the face pictures; the dot remained on the screen until a response was made (see Fig. 3). Participants were instructed to press a button corresponding to the side where the dot appeared as quickly as possible using both index fingers. We established two conditions, congruent and incongruent, based on the combination of places where the emotional face and dot appeared: the dot appeared either on the same (congruent) or the opposite (incongruent) side as the emotional face; each condition was displayed with equal probability. Furthermore, the emotional faces (happy, sad, and angry) were displayed on the left or right side with equal probability, in a pseudo-random order. The software recorded participants’ response accuracy and latency.

#### Emotional Stroop task

The stimulus words included 10 each of sad, threatening, and neutral words. This word set was created by referring to previous reports using the same task^45,46^, and then translating each word into Japanese two-kanji compound words. Participants rated the words on a separate day from the main experiment; participants were asked to rate the fearfulness and sadness of each word using a 7-point Likert-type scale ranging from 1 (*not sad or fearful at all*) to 7 (*very sad or fearful*). A two-way (word category × rating [sadness and fearfulness]) repeated-measures ANOVA on the ratings showed a statistically significant main effect of word category {*F*(1.686, 38.787) = 309.358, *P* = 0.000} and interaction {*F*(1.546, 35.550) = 69.701, *P* = 0.000}, however, the main effect of rating was not significant. Post-hoc multiple comparisons (Bonferroni-corrected) revealed that the word ratings of the each word category differed significantly: the sadness score of the sad words (5.42 ± 0.22) was significantly higher than the fearfulness score of those (4.25 ± 0.51; *P* = 0.000); the fearfulness score of the threatening words (5.75 ± 0.71) was significantly higher than the sadness score of those (4.53 ± 0.69; *P* = 0.000); likewise, the sadness score of the sad words was significantly higher than that of the threatening words (*P* = 0.000); the fearfulness score of the threatening words was significantly higher than that of the sad words (*P* = 0.000). The sadness score and fearfulness score of the neutral words were 1.38 ± 0.24 and 1.53 ± 0.29, respectively.

**Figure 4:**
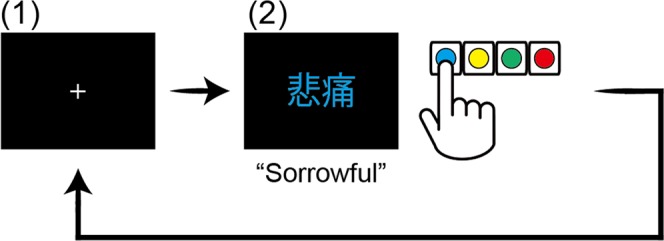
Emotional Stroop task procedure. (1) A white fixation cross was presented at the centre of the screen for 500 ms. (2) Two-kanji compound words coloured blue, yellow, green, or red were displayed until participants made a response. Participants pressed the button corresponding to the colour of the word as quickly as possible without interpreting the word’s meaning.

In this task, the words were coloured blue, yellow, green, or red, and displayed on a black background in a pseudo-random order. As with the FDP task, each trial began with a white fixation cross presented at the centre of the screen for 500 ms, after which a word appeared (see Fig. 4). The word remained until participants made a response. Participants were instructed to respond as quickly as possible by pressing a button corresponding to the colour of the word using their right index, middle, ring, and little fingers. They were asked to pay attention only to the colour of the word and ignore its meaning.

### Data analysis

All the statistical analyses were performed using SPSS Statistics 23.0 (IBM Corp., Armonk, NY, USA). *P* values of less than 0.05 were considered statistically significant. All *P* values in the present study were two-tailed. To estimate the power of the tests in the present study, effect sizes were calculated. We chose the partial eta-squared (η_p_^2^) as the estimate of effect size for the main effects and interaction of the ANOVA, and Hedge’s *g* for post-hoc multiple comparisons of the ANOVA and the paired samples *t*-test. Hedge’s *g* is more accurate than Cohen’s *d* when the sample size is relatively small^24,47^. The design of the present study was dependent (paired), so that we used the equation advocated by Dunlap *et al*.^48^ to calculate Cohen’s *d*, which was

$$d=t{[2(1-r)/n]}^{1/2}$$

where *t* was the *t* statistic from paired samples *t*-test and *r* was the correlation across the pairs of measures. To convert Cohen’s *d* into Hedge’s *g*, we used a correction factor, called *J*^49^:

$$J=1-3/(4df-1),$$

$$g=J\times d.$$

The magnitude of the effect is commonly assessed using the thresholds provided by Cohen^50^: |*g*| < 0.2, negligible; |*g*| < 0.5, small; |*g*| < 0.8, medium; |*g*| ≥ 0.8, large. We excluded data from three individuals because they had correct answer rates on the two-back task that were not significantly above chance.

#### Two-back task

In the analyses, we used the following as variables: mean reaction time of all sessions and each session; the correct answer rate, incorrect answer rate, and unanswered rate of all sessions and each session; and the VAS scores.

#### Face dot-probe task

We excluded all incorrect trials and, in order to avoid the influence of outliers, trials with reaction times of less than 100 ms or greater than 1000 ms^19^. ‘Face attentional bias score’ was defined as the mean reaction time in the incongruent condition subtracted by the mean reaction time in the congruent condition^22^. When face attentional bias score is greater than zero, the participant pays attention more on the emotional stimuli than neutral: they show a ‘vigilant’ attentional bias toward the emotional stimuli; when the score is less than zero, the participant focuses less on the emotional stimuli: they show an ‘avoidant’ attentional bias for the emotional stimuli. We calculated this score for each condition, and then analysed differences in this score via a repeated measures ANOVA with emotion (happy, sad, and angry) and timing (pre- and post-fatigue) as factors.

#### Emotional Stroop task

As with the FDP task, incorrect trials and the trials with outlying reaction times (less than 100 ms or greater than 1500 ms) were excluded from the analysis. We calculated a ‘word attentional bias score’ as the mean reaction time in the emotional (threatening or sad) word condition subtracted by the mean reaction time in the neutral word condition. As with face attentional bias score, word attentional bias score greater than zero could be interpreted as the participant shows a ‘vigilant’ attentional bias toward the emotional stimuli; the score less than zero meant that they show an ‘avoidant’ attentional bias for the emotional stimuli. This score was analysed via a repeated measures ANOVA with emotion (sad and threatening) and timing (pre- and post-fatigue) as factors.
